# Integrated community case management (iCCM) of childhood infection saves lives in hard-to-reach communities in Nicaragua

**DOI:** 10.26633/RPSP.2017.66

**Published:** 2017-04-14

**Authors:** Dixmer Rivera, Rashed Shah, Tanya Guenther, Meredith Adamo, Jeanne Koepsell, Carmen Maria Reyes, Mary McInerney, David R Marsh

**Affiliations:** 1 Save the Children International Save the Children International Managua Nicaragua Save the Children International, Managua, Nicaragua.; 2 Save the Children USA Save the Children USA Washington, DC United States of America Save the Children USA, Washington, DC, United States of America.; 3 Warren Alpert Medical School, Brown University, Providence Warren Alpert Medical School, Brown University, Providence ProvidenceRhode Island United States of America Warren Alpert Medical School, Brown University, Providence, Rhode Island, United States of America.; 4 Retired Senior Advisor, Child Health, Save the Children Retired Senior Advisor, Child Health, Save the Children AmherstMassachusetts United States of America Retired Senior Advisor, Child Health, Save the Children, Amherst, Massachusetts, United States of America.

**Keywords:** Community health workers, case management, primary health care, infant mortality, Nicaragua, Agentes comunitarios de salud, manejo de caso, atención primaria de salud, mortalidad infantil, Nicaragua, Agentes comunitários de saúde, administração de caso, atenção primária à saúde, mortalidade infantil’, Nicaragua

## Abstract

**Objective.:**

To describe Nicaragua’s integrated community case management (iCCM) program for hard-to-reach, rural communities and to evaluate its impact using monitoring data, including annual, census-based infant mortality data.

**Method.:**

*This observational study measured the strength of iCCM implementation and estimated trends in infant mortality during 2007–2013 in 120 remote Nicaraguan communities where brigadistas (“health brigadiers”) offered iCCM services to children 2–59 months old. The study used program monitoring data from brigadistas’ registers and supervision checklists, and derived mortality data from annual censuses conducted by the Ministry of Health. The mortality ratio (infant deaths over number of children alive in the under-1-year age group) was calculated and point estimates and exact binomial confidence intervals (CIs) were reported*.

**Results.:**

Monitoring data revealed strong implementation of iCCM over the study period, with medicine availability, completeness of recording, and correct classification always exceeding 80%. Treatments provided by brigadistas for pneumonia and diarrhea closely tracked expected cases and caregivers consistently sought treatment more frequently from brigadistas than from health facilities. The infant mortality ratio decreased more in iCCM areas compared to the non-iCCM areas. Statistically significant reduction ranged from 52% in 2010 (mortality rate ratio 0.48; 95% CI: 0.25–0.92) to 59% in 2013 (mortality rate ratio 0.41; 95% CI: 0.21–0.81).

**Conclusions.:**

The iCCM has been found to be an effective and feasible strategy to save infant lives in hard-to-reach communities in Nicaragua. The impact was likely mediated by increased use of curative interventions, made accessible and available at the community level, and delivered through high-quality services, by brigadistas.

## RESUMEN

**Objetivo.:**

Describir el programa nicaragüense de atención integrada en la comunidad destinado a las comunidades rurales de difícil acceso y evaluar sus efectos a partir de los datos de seguimiento, como los datos censales anuales sobre la mortalidad infantil (menores de 1 año).

**Método.:**

Este estudio de observación tuvo como objetivo valorar la ejecución de la atención integrada en la comunidad y medir las tendencias de la mortalidad infantil (menores de 1 año) entre el 2007 y el 2013 en 120 comunidades nicaragüenses remotas donde los brigadistas brindan servicios de atención integrada en la comunidad a niños de 2 a 59 meses. El estudio se valió de datos programáticos de seguimiento obtenidos de los registros y las listas de verificación utilizadas para la supervisión de los brigadistas, así como datos de mortalidad derivados de los censos anuales realizados por el Ministerio de Salud. Se calculó la razón de mortalidad (defunciones de lactantes por cantidad de menores de un año vivos) y se informaron estimaciones puntuales e intervalos de confianza binomiales exactos.

**Resultados.:**

Los datos de seguimiento mostraron una ejecución sólida del programa de atención integrada en la comunidad en el período del estudio, dado que la disponibilidad de medicamentos, los registros completos y la clasificación correcta siempre superaron el 80%. Como parte del tratamiento proporcionado contra la neumonía y la diarrea, los brigadistas llevaron a cabo un seguimiento minucioso de los casos previstos y los cuidadores solicitaron tratamiento de los brigadistas con más frecuencia que de los establecimientos de salud. La razón de mortalidad infantil (menores de 1 año) disminuyó más en las zonas atendidas por el programa que en aquellas no atendidas. Se observó una disminución estadísticamente significativa del 52% en el 2010 (razón de la tasa de mortalidad 0,48; IC del 95%: 8%-75%) al 59% en el 2013 (razón de la tasa de mortalidad 0,41; IC del 95%: 19%-79%).

**Conclusiones.:**

El programa de atención integrada en la comunidad es una estrategia eficaz y factible para salvar la vida de los lactantes en las comunidades de difícil acceso de Nicaragua. Estos efectos probablemente estuvieron mediados por la mayor prestación de intervenciones curativas, que estuvieron accesibles y disponibles en el ámbito de la comunidad, y fueron brindadas por los brigadistas por medio de servicios de buena calidad.

## RESUMO

**Objetivo.:**

Descrever o programa de manejo integrado de casos na comunidade (iCCM) para comunidades rurais e de difícil acesso na Nicarágua e avaliar seu impacto usando dados de monitoramento, inclusive dados censitários anuais de mortalidade infantil.

**Método.:**

Este estudo observacional avaliou o nível de implementação da estratégia iCCM e calculou tendências de mortalidade infantil nos anos 2007–2013 em 120 comunidades remotas da Nicarágua onde “brigadistas de saúde” prestam serviços de iCCM para crianças de 2 a 59 meses de idade. Foram utilizados dados de monitoramento obtidos dos cadastros e checklists de supervisão de brigadistas e dados de mortalidade obtidos dos censos anuais realizados pelo Ministério da Saúde. Foi calculado o coeficiente de mortalidade infantil (número de óbitos infantis dividido pelo número de crianças menores de 1 ano vivas), além de estimativas pontuais e intervalos de confiança (IC) binomiais exatos.

**Resultados.:**

Os dados de monitoramento evidenciaram forte implementação da estratégia iCCM durante o período de estudo; a disponibilidade de medicamentos, a integralidade dos registros e a classificação correta sempre excederam 80%. O tratamento da pneumonia e da diarreia por brigadistas acompanhou de perto o número de casos esperado, e os responsáveis pelas crianças sistematicamente buscaram tratamento com maior frequência junto aos brigadistas do que nos serviços de saúde. O coeficiente de mortalidade infantil diminuiu mais nas áreas com iCCM do que nas áreas sem iCCM. A redução foi estatisticamente significante, variando de 52% em 2010 (razão de taxa de mortalidade: 0,48; IC95%: 8%–75%) a 59% em 2013 (razão de taxa de mortalidade: 0,41; IC95%: 19%–79%).

**Conclusões.:**

Constatou-se que a iCCM é uma estratégia eficaz e viável para salvar vidas de crianças em comunidades de difícil acesso na Nicarágua. O impacto provavelmente foi mediado pelo aumento do uso de intervenções resolutivas, disponibilizadas de maneira acessível no âmbito da comunidade, através do serviço qualificado prestado pelos brigadistas.

Nicaragua is on track to achieve its target for Millennium Development Goal (MDG) 5 (1), having reduced its mortality rate for children under 5 years old from 66 to 24 deaths per 1 000 live births, with a 4.5% per year reduction during 1990–2012 (2). According to recent estimates from the UNICEF global database for under-5 mortality (2015), the leading causes of death in children under 5 in Nicaragua are 1) congenital conditions (20.4%); 2) preterm conditions (17.8%); 3) other conditions (17.1%); 4) pneumonia (16.2%); 5) diarrhea (8.1%); 6) intrapartum complications (7.7%); 7) injury (5.5%); and 8) sepsis (3.9%) (3). However, subnational mortality disparities persist against rural and poor residents, who have geographic, social, and economic barriers to health care access (4). Nicaragua’s most recent Demographic and Health Survey (DHS) (*Encuesta Nicaragüense de Demografía y Salud 2011/12*) revealed rural–urban disparities in the prevalence of acute respiratory infections (30.0% versus 27.0%) and diarrhea (16.0% versus 14.8%); the use of oral rehydration solution (ORS) to treat childhood diarrhea (57.0% versus 74.2%); and care-seeking for possible pneumonia (72.3% versus 85.3%) (5).

Death from common childhood infectious diseases (e.g., diarrhea and pneumonia) is avoidable if families can reach and use services that deliver evidence-based curative interventions. In rural areas this is achievable through integrated community case management (iCCM), a health care delivery strategy that 1) selects, trains, supplies, deploys, supports, and supervises resident community health workers (CHWs) to treat sick children whose families lack access to health facilities and 2) mobilizes families to seek prompt and appropriate care (6).

Nicaragua groups rural communities into three categories according to their geographic access to a health facility: “A” (< 1 hour from a facility); “B” (1–2 hours away); and “C” (> 2 hours away). About one-third (30%) of rural communities are designated as “category C” (approximately 1 500). At-risk populations—specifically women and children—in category C communities are vulnerable to inadequate or delayed treatment, advanced disease, or death, due to distance from a health facility, seasonal road impassibility, lack of public transport, and cost (7). The Nicaraguan Ministry of Health (MINSA) introduced iCCM in 2006 and gradually scaled it up, with partners’ support, to serve 175 category C communities in 32 *municipios* (districts) in the departments (provinces) of Chontales, Jinotega, Léon, Matagalpa, and Río San Juan, and in the Atlantic regions. The iCCM strategy includes treatment delivery to sick children 2–59 months old through existing volunteer CHWs known as *brigadistas* (8).

Mortality studies within programs are costly and challenged by insufficient sample sizes, incomplete mortality data, weak implementation strength, and imperfect comparisons. The available iCCM evidence base relies on mortality reduction from efficacy studies in highly controlled settings (9). Given that only one trial (in Ghana) out of seven effectiveness studies conducted in Africa (Burkina Faso, Cameroon, Ethiopia, Ghana, Sierra Leone, Uganda, and Zambia) showed statistically significant mortality reduction due to iCCM, likely due to the challenges noted above, the 2014 Global iCCM Evidence Review Symposium (held in Accra) recommended systematic, in-depth use of monitoring and utilization data (9, 10). The purpose of this study was to describe Nicaragua’s iCCM program for hard-toreach, rural communities and to evaluate its impact using monitoring data, including annual, census-based infant mortality data.

## Program description

MINSA, with support from Save the Children (Managua), introduced iCCM in Nicaragua through the local comprehensive health care system and its personnel (*Sistemas Locales de Atención Integral de Salud*, SILAIS) and implemented the current iCCM program through eight health system components: 1) organization, coordination, and policy setting; 2) human resources; 3) service delivery and referral; 4) behavior and social change, sensitization, and advocacy; 5) supply chain management; 6) recruitment, training and supervision; 7) monitoring, health management information system (HMIS), evaluation, and research; and 8) budgeting, costing, and financing (11, 12).

### Organization, coordination, and policy setting

Nicaragua’s Program for Community Health and Nutrition (*Programa Communitaria de Salud y Nutricion,* PROCOSAN) supported a platform for community maternal and child health interventions with the aid of *brigadistas* (4, 13). Infant and child health strategies under PROCOSAN include growth monitoring and nutrition counseling; detection of early warning signs of illness, and counseling on home-based management of childhood illness; referrals to health facilities, and follow-up; and provision of vitamin A supplements and immunization. The PROCOSAN Technical Operations Manual also included iCCM as a component of its programming. MINSA sets strategic direction for planning and implementing iCCM through SILAIS personnel and *brigadistas*, with support from implementing partners such as Save the Children. The policy initially forbade iCCM, then allowed it pending national evidence, and currently specifies it as the national norm for category C communities (14). An iCCM Technical Advisory Group reviewed and endorsed the strategy specified in the Technical Operations Manual.

### Human resources.

In Nicaragua, *brigadistas* deliver the iCCM services at the community level. The *brigadista* concept was developed in 1980, when the Nicaraguan government assigned 100 000 young Nicaraguan volunteers to facilitate an intensive literacy campaign (15). In 1981 MINSA began training its own CHWs, known as *brigadistas de salud* (“health brigadiers”), to strengthen community participation in health and expand health education nationwide (16). Save the Children supported 360 *brigadistas* in the delivery of PROCOSAN to 120 communities in 15 municipalities in the departments of Jinotega, León, and Matagalpa. More details about *brigadistas* in Nicaragua are provided by Adamo et al. (17). The iCCM was implemented by an integrated team consisting of *brigadistas*, local health center and health post nursing staff, Save the Children field supervisors, and SILAIS supervisors at the department (province) level. *Brigadistas* assessed and classified illnesses, administered treatment, counseled caregivers, facilitated referral for serious illness, and made follow-up visits to the homes of all treated children. Health post nurses supervised *brigadistas*, received referrals, and issued back-referrals. Health center personnel served as municipal-level coordinators of iCCM, training health post nurses to oversee *brigadista*s; ensured a constant supply of medicines; participated in supervision; and reported results to SILAIS managers. At the department level, SILAIS managers monitored the iCCM program to ensure the rational use of medicines and take account of its overall impact.

### Service delivery and referral

**FIGURE 1:**
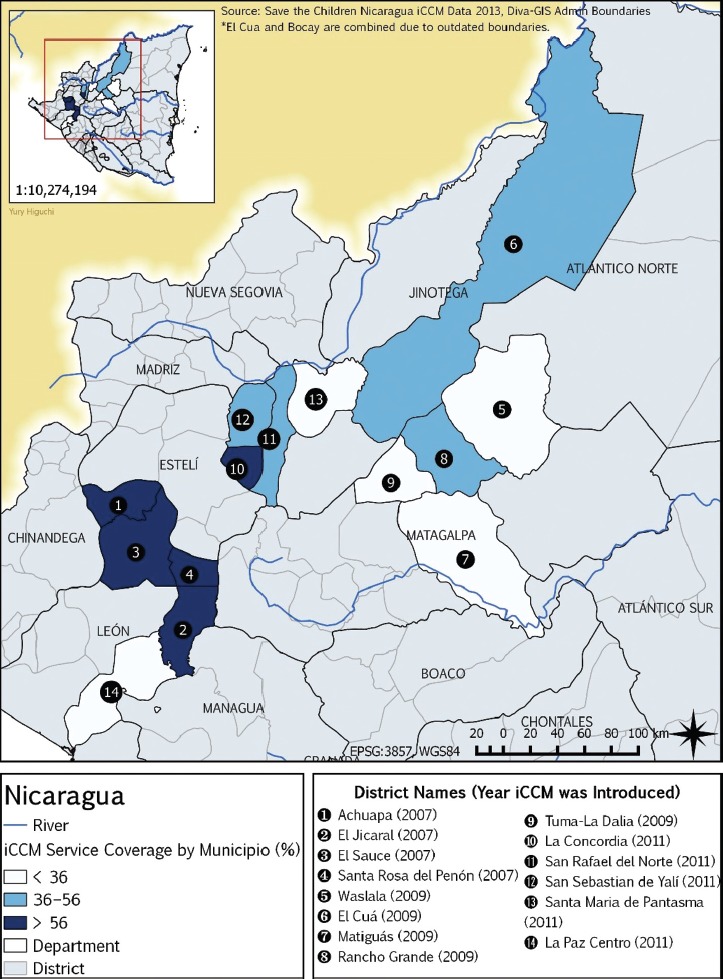
Integrated community case management (iCCM) service coverage (%) by district (municipio), Nicaragua, 2007–2013

***Source:*** Prepared by the authors based on data from the Save the Children iCCM program in Nicaragua

Communities were selected for iCCM service delivery based on low access (i.e., category C designation by MINSA) and feasibility (i.e., within close proximity for supervision, supply, and referral). The program area map is shown in Figure 1. The iCCM services included treatment for pneumonia (oral amoxicillin), diarrhea (ORS and zinc), and dysentery (furazolidone), plus paracetamol for fever. The color-coded case management guide (*Guía de Abordaje*) was an adaptation of the Integrated Management of Childhood Illness flowchart (18) that health workers still use in the clinics. *Brigadistas* facilitated referral for severe disease with pre-referral treatment, a referral note, and local problem-solving to assure compliance. They recorded each case in a treatment register, specifying the date, the name, and tick-based choices for age group, classification, treatment, referral, and compliance with referral or treatment. *Brigadistas* gave a purple handkerchief (*bandera*) to mothers when referring cases to health posts or higher centers. A handkerchief on the child’s head reminded receiving facility staff that the patient was a complicated case, referred by *brigadistas*, necessitating immediate attention.

### Behavior and social change, sensitization, and advocacy.

Communities nominated resident *brigadistas,* specifying one to manage the medicine kit (*botiquín*)*.* Every *brigadista* mobilized the community for appropriate and prompt care-seeking through group health talks, home visits, and community meetings. During a home illness visit, *brigadistas* counseled the mother and family about supportive home care and treatment. They also obtained the caregiver’s signature on a “commitment reminder” (*recordatorio de compromiso)* that pictorially illustrates age-specific doses for all medicines, which the caregivers marked and presented to *brigadistas* for review during follow-up visits.

### Supply chain management.

All iCCM medicines were on the national Essential Medicines List and MINSA ensured an uninterrupted supply. *Brigadistas* who received iCCM training were deployed with a *botiquín* stocked with a two-month supply of medicines. The *botiquín* included dissolvable zinc tablets and ORS, furazolidone suspension, amoxicillin syrup, acetaminophen syrup, a one-liter container to mix the ORS, counseling cards, commitment reminders, soap, cotton balls, clean towels, a thermometer, a timer to count respirations, and a spoon. *Brigadistas* also received name tags, a raincoat, and flashlights (for home visits). Thereafter, a health worker or Save the Children field technician (*técnico*) delivered MINSA medicines to each community monthly in accordance with seasonally expected pneumonia and diarrhea cases, cross-checking use against recorded treatments in the register. When outbreaks occurred or when the weather might not permit the next scheduled visits, the *brigadistas* received more drugs than the official norm.

### Recruitment, training and supervision

Save the Children supported training for both *brigadistas* and MINSA supervisory staff to deliver iCCM in the defined program area, which had a total population of 6 870 children under 5 years old by the end of the study (2013). *Brigadistas* received training on a variety of health information and service activities and were supported and supervised by health post nurses. All iCCM *brigadistas* were trained as PROCOSAN *brigadistas* in a 14-day training session (five days for community health promotion, six days for birth planning and family planning, and three days for morbidity). In addition, the iCCM *brigadistas* completed an initial 20-hour training session (over 3 days). The iCCM *brigadista* training was supplemented by monthly supervision visits and two-day refresher training at six-month intervals. Several iCCM *brigadistas* from the same community received the three-day training, but only one—the principal provider—received the fully stocked *botiquín*, supported by another type of *brigadistas* known as *asistentes* (assistants) in follow-up visits or during absences. Health center and health post staff were trained for three days in iCCM interventions and for one day on support and supervision, using the Technical Operations Manual (*Manual Técnico Operativo Salud y Nutrición en la Niñez*). Each *brigadista* received a *brigadista* manual (*Manual del Brigadista*); health post staff received a training manual (*Guía de Capacitación*) plus a *brigadista* manual for reference.

Most health post nurses supported three or more iCCM sites, each with three *brigadistas*. Health post nurses supervised *brigadistas* every 1–2 months using a supervision checklist (*lista de apoyo a la supervision*) to assess performance on 1) availability of forms, supplies, and medicines; 2) correct application of a decision tree (for classification and diagnosis); 3) appropriate selection of treatment; and 4) counseling. The health center nurse accompanied the health post nurse on approximately half the supervisory visits. Supervisors (MINSA health staff from the municipal SILAIS and Save the Children *técnicos*) coached *brigadistas* for any identified deficiencies. The rare case of persistent nonperformance resulted in surrendering the *botiquín* to another *brigadista*. A nurse manager, epidemiologist, or *municipio* educator supervised the supervisors 4–6 times annually, using the same checklist.

### Monitoring, HMIS, evaluation, and research.

Activities conducted by supervisors during a supervisory visit included reviewing treatment registers; observing *brigadistas*’ care of sick children or response to a hypothetical case^5^ (“case scenario”); comparing treatments recorded in the register with the remaining stock; and reviewing home visits, referrals, and patient compliance with treatment and referral. Supervisors awarded a “pass” if the register was complete and consistent for availability of both antibiotics and ORS on the day of supervision. For classifying and counseling, a “pass” was awarded for a perfect score (10 out of 10) in three out of a maximum of five case scenarios (actual and hypothetical). A monthly review was conducted to 1) detect changes over time and between different catchment areas and 2) provide feedback at community progress review meetings held every four months.

### Budgeting, costing, and financing.

Save the Children prepared a budget for the initial 14-community pilot study. MINSA provided most medicines and staff for the pilot and the first study expansion, while Save the Children provided technical support and funding for training materials, training, *brigadistas*’ supplies, and zinc sulfate. Save the Children also performed a funding gap analysis for each phase of the project. MINSA continued to pay costs for staff, medicines, and training of *brigadista* in approximately 500 of the 1 500 category C communities.

## MATERIALS AND METHODS

### Study design

This study had an observational design that included measurement of 1) the strength of iCCM implementation over time in the study areas and 2) estimated trends in mortality, comparing areas with iCCM to areas without iCCM as the program expanded.

### Data sources

This study used program monitoring data from *brigadista*s’ treatment registers and supervision checklists (*lista de apoyo a la supervisión*). Monitoring data were submitted to the municipal health center nurse who reviewed and computerized all iCCM data (monthly) and calculated the indicators (e.g., number of children treated by age, number of treatment courses provided by type, referrals completed, *brigadistas*’ adherence to iCCM protocol, etc.) for reporting to the department and SILAIS.

#### Program mortality data.

Nicaragua lacks complete vital registration for category C communities. However, PROCOSAN required an annual census of its program communities (with or without iCCM), conducted in the same month each year (varying by department), which yielded 38 indicators. After training from senior MINSA staff (followed up with biannual refresher training), health post workers visited each household covered by the PROCOSAN (preventive) program and asked the mother 17 questions from a large register (8). The census teams then revisited households as necessary, aiming for 100% participation. The census recorded, among other variables, names and birthdates of all living children under 2 years old and the details of any deaths that had occurred in the past 12 months in that age group.

### Statistical analyses

The following variables related to implementation strength (19) were defined and assessed for study areas with iCCM: 1) access, 2) human resources, 3) supply chain, 4) supervision and quality, and 5) utilization (Table 1). In the absence of birth histories, annual infant mortality rates were approximated using the number of living children less than 1 year old as the denominator (rather than live births) and the number of children dying before they reached 1 year of age as the numerator. The numerator was derived by scanning a line list^6^ of reported deaths in children under 2 years old and identifying those dying before they were 1 year old. The ratio of deaths in children under 1 year old to the number of living children under 1 year old (i.e., the crude mortality ratio) was calculated along with point estimates and exact binomial confidence intervals (CIs) for each year. The *ptrend*^7^ command in Stata^®^ (20) was used to calculate the chi-square statistic to test the significance of trends over time in quality of care indicators between 2007 and 2013.

### Ethics

Ethical approval from an institutional review board was not required for this study because of its data source (program monitoring data, including census data that were not linked to any individual human subject). MINSA confirmed that it had no objection to the study.

## RESULTS

This study recorded a total of 243 infant deaths and 7 564 live infants, based on PROCOSAN annual censuses during 2007–2013. The iCCM program targeted 26.6% (17 out of 64) of category C communities that had PROCOSAN’s nutrition component at the beginning of the study (2007) and 46.2% (120 out of 260) by the end of the study (2013), with continuous availability of 100% of iCCM services in all targeted communities throughout the study period. *Brigadista* density was about 1 per 57 children under 5 years of age. The quality of iCCM services was high throughout the seven-year study period (Table 1). Availability of medicines and completeness of recording always exceeded 90%. Correct classification always exceeded 80% and improved further over time. The percentage of counseling coverage started out relatively low (70.0%) but improved by 27.0 percentage points over the study period. The only demand indicator available from the program monitoring data was completion of the recommended treatment, which exceeded 95.0% starting in 2008, when those data were first available. Caregivers consistently sought treatment for childhood pneumonia or diarrhea more frequently from *brigadistas* than from a health facility. *Brigadistas* and their facility-based counterparts— with a few exceptions—treated more cases of pneumonia than diarrhea.

**TABLE 1:** Strength of integrated community case management (iCCM) program implementation by year, Nicaragua, 2007–2013

Outcome variable	Indicator	Indicator definition	Service coverage (%)						
			2007	2008	2009	2010	2011^a^	2012	2013
Access	Community coverage	No. of “category C”^b^ communities with one or more *brigadistas* trained in iCCM and equipped with a medicine kit (*botiquín*)/no. of targeted category C communities	6.5	17.3	28.5	32.7	45.8	46.2	46.2
Human resources	Density of service delivery points (per 1 000 children under 5)	No. of service delivery points/no. of children under age 5 in targeted category C communities	13.1	19.9	16.2	12.4	17.3	17.5	17.5
Supply chain	Medicine availability	No. of service delivery points with all iCCM medicines available during a supervision day/no. of service delivery points supervised	95.0	95.0	91.0	90.1	91.0	93.4	92.7
Supervision and quality	Supervision visits per year^c^	No. of supervision visits conducted/no. of expected supervision visits based on protocol	87.7	85.9	86.7	91.7	90.8	91.7	91.3
	Counseling^c^	No. of *brigadistas* who provide correct counseling during an actual sick child consultation or hypothetical case presented by the supervisor (“case scenario”) at time of supervision/no. of *brigadistas* who responded to an actual/hypothetical case scenario during supervision	70.0	79.0	86.0	90.0	95.5	96.7	97.0
	Correct classification^c^	No. of *brigadistas* who correctly classify a sick child or hypothetical case (“case scenario”) at time of supervision/no. of *brigadistas* who responded to an actual/real case scenario during supervision	83.0	93.0	95.0	89.1	90.0	91.3	91.8
	Recording completion	No. of cases registered by *brigadistas* with complete information/no. of total cases registered by *brigadistas*	95.0	93.0	95.0	94.8	90.0	94.7	95.3
	Treatment completion^c^	No. of cases treated by *brigadistas* in which mother reportedly completed treatment/no. of cases treated by *brigadistas*	96.3	98.0	95.0	96.0	96.1	98.4	98.4
Utilization	Caseload by community	No. of cases treated annually by *brigadistas /* no. of category C communities with one or more *brigadistas* trained in iCCM and equipped with a medicine kit (*botiquín*)	45.9	26.9	31.8	39.6	28.0	22.6	26.6
	Treatment contribution of *brigadistas*	No. of cases treated annually by *brigadistas* (for both pneumonia and diarrhea)/no. of cases treated by health facilities and *brigadistas*	83.3	78.9	78.2	75.5	75.3	72.7	81.1

***Source:*** Prepared by the authors based on the study data.

a Year of introduction of the pneumococcal conjugate vaccine.

b Nicaraguan Ministry of Health designation for the ∼30% of rural communities nationwide that are > 2 hours away from a health facility.

c *P*-value for the trend analysis was significant (< 0.01).

**FIGURE 2.**
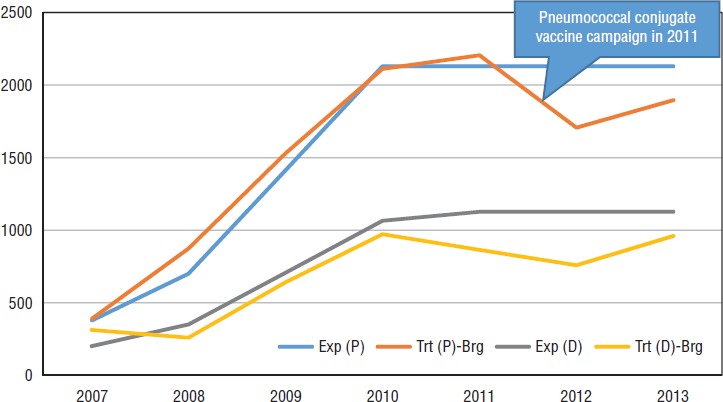
Pneumonia (“P”) and diarrhea (“D”) in children under 5 years old: expected (“Exp”) cases^a^ versus cases treated (“Trt”) by *brigadistas* (“-Brg”), 2007–2013^b^

***Source:*** Prepared by the authors based on data from the Save the Children integrated community case management (iCCM) program in Nicaragua.

^a^ Established by MINSA based on 1) Nicaragua’s Demographic and Health Survey (DHS) 2006–2007 (21), which reported pneumonia/diarrhea prevalence as 29.1%/15.5%, and 2) contemporary research (Rudan et al. (2008), for 2008–2013, who reported 0.31 pneumonia episodes per child per year (22), and Becker-Dreps et al. (2011) for 2011–2013, who reported 0.164 diarrheal episodes per child per year (23)).

^b^ The population of children under 5 served by iCCM increased from 1 296 in 2007 to 6 870 in 2013.

Actual utilization generally tracked expected utilization (i.e., treatment ratios), often closely (Figure 2). For example, *brigadistas* treated 97.3% of expected pneumonia cases between 2007 and 2013 compared to 127.1% by *brigadistas* plus health facilities; the comparable numbers for diarrhea treatment were 83.6% versus 115.3% (data not shown). The number of expected cases was established by MINSA based on 1) DHS 2006– 2007 (21), which reported pneumonia and diarrhea prevalence as 29.1% and 15.5% respectively, and 2) contemporary research (Rudan et al. (2008), for 2008– 2013, who reported 0.31 pneumonia episodes per child per year (22), and Becker-Dreps et al. (2011), for 2011–2013, who reported 0.164 diarrheal episodes per child per year (23)).

**FIGURE 3.**
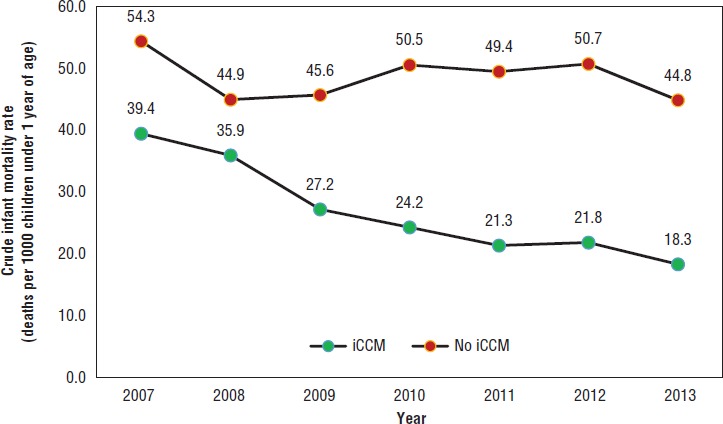
Crude infant mortality rate (estimated deaths per 1 000 children under 1 year old)^a^ in category C communities,^b^ by year and integrated community case management program status (“iCCM” versus “No iCCM”), Nicaragua, 2007–2013

***Source:*** Prepared by the authors based on the study data.

^a^ Numerator and denominator from annual census sweeps of communities with a preventive program (PROCOSAN) in the departments of Jinotega, León, and Matagalpa.

^b^Nicaraguan Ministry of Health designation for the ∼30% of rural communities nationwide that are > 2 hours away from a health facility.

**FIGURE 4.**
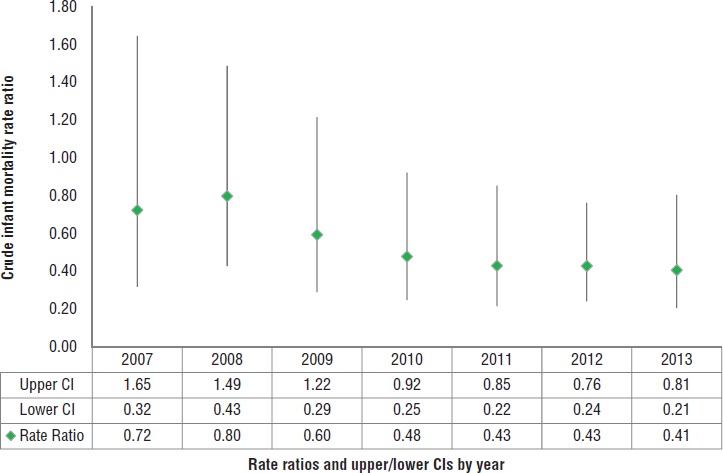
Crude infant mortality rate ratios^a^ and confidence intervals (CIs) in category C communities^b^ with and without iCCM, by year, Nicaragua, 2007–2013

***Source:*** Prepared by the authors based on the study data.

^a^ Estimated crude infant mortality rate in integrated community case management (iCCM) areas (numerator) over estimated crude mortality rate in non-iCCM areas (denominator).

^b^ Nicaraguan Ministry of Health designation for the ∼30% of rural communities nationwide that are > 2 hours away from a health facility.

Figure 3 shows infant mortality (deaths per 1 000 living infants) for iCCM and non-iCCM areas during 2007–2013. The infant mortality ratio decreased more in iCCM areas than in non-iCCM areas. The mortality level in category C communities with iCCM was significantly less than in category C communities without iCCM beginning in 2010 (rate ratio: 0.48; 95% CI: 0.25–0.92) (Figure 4) and persisting through the end of the study period (2013).

## DISCUSSION

The results of this study revealed strong iCCM implementation in target communities in Nicaragua, most likely facilitated by high utilization of the highly accessible, high-quality, and highly demanded and enabled iCCM services. Given this high level of utilization, it was not surprising that mortality among infants decreased. What was surprising was the success of a method that included the use of highly affordable program monitoring data.

These results are consistent with others’ findings that iCCM increases access to effective treatment at the community level (24, 25) while decreasing the workload at primary health care centers (26, 27). Another positive study finding was that care-seekers completed the recommended treatment. An earlier evaluation noted that caregivers cited two program components that helped ensure compliance: 1) the *recordatorio de compromiso*, which listed age-specific doses for the prescribed medicines, and was marked and presented to a *brigadista* for review, and 2) the *brigadista’s* personal follow-up and counseling (4).

These data also indicated a drop-off in pneumonia treatment in 2012, which was assumed to correlate with the countrywide introduction of the pneumococcal conjugate vaccine in 2011. Other possible explanations for the lower incidence that year for pneumonia, and diarrhea, include 1) program maturation, with better illness classification; 2) increased use of other preventive interventions (i.e., nationwide campaigns for the pneumococcal and rotavirus vaccines, zinc supplementation, vitamin A supplementation, and deworming); and 3) an unusually mild winter. In addition, improved water supply and sanitation, and handwashing (possibly resulting from awareness campaigns about handwashing during an outbreak of influenza A virus subtype H1N1 in 2009) could have contributed to disease reduction.

This study adds value to the evidence base for impact of iCCM on reducing child mortality and, to the best of the authors’ knowledge, is the first research to report mortality reduction achieved through integrating iCCM within an ongoing community-based health and nutrition program in a non-African or non-Asian setting. The results, which include comparative estimates of infant mortality in iCCM and non-iCCM areas in category C communities, show that the latter areas had higher mortality rates (although the differences were not statistically significant) at the beginning of the study (2007), possibly due to their hard-to-reach locations, which may have restricted any type of program implementation. After three years of program delivery (by 2010), the iCCM areas had lower infant mortality compared to the non-iCCM areas (with statistically significant differences). The reduction in infant deaths observed in this study (a drop of 59.3%) was consistent with the potential reduction (63%) in children under age 5 reported in a systematic review of national CHW programs in sub -Saharan Africa with curative interventions for childhood malaria, pneumonia, and diarrhea (28). Another study by Mugeni et al. (29) reported a 38% reduction of mortality in children under 5 years old in Rwanda after introducing iCCM nationwide.

### Limitations

One limitation of this study was that full birth histories, which are recommended for evaluating mortality impact, could not be obtained. However, in the authors’ view that limitation was counterbalanced by the use of the program monitoring data collected by PROCOSAN in its annual surveys of its coverage areas. Although in some settings, especially those with large numbers of supervisors with weak oversight, the use of routinely collected supervision data has been shown to result in overestimation of certain aspects of program quality (e.g., the ability of CHWs to accurately count respiratory rates) (24), in the iCCM program studied here, well-trained and well-managed supervisors collected the monitoring data. Therefore, the authors believe the use of program monitoring data, as recommended by an iCCM symposium held in Accra in 2014 (10, 30), strengthened this study. For example, given the lack of alternative sources of care in category C communities, any cross-contamination (i.e., care-seeking by residents of communities without iCCM in communities with iCCM) would tend to mask differences between the two types of patient pools, but in this study, the differences persisted.

Another limitation of this study was the lack of household or health service surveys to triangulate the findings from routine data or to compare differences in overall treatment levels between iCCM and non-iCCM areas, and over time. However, the use of data on actual treatments given (versus reported treatments from surveys) and their comparison with expected levels of disease, as carried out in this study, provides good evidence of high coverage and at much lower cost.

### Conclusions

Despite a study design that constrained direct attribution of this infant mortality reduction to implementation of iCCM, the findings are in line with those of prior studies conducted in other low-income countries. The results of the study support iCCM as an effective, feasible strategy to save infant lives in hard-to-reach communities in Nicaragua. This report also provides details on Nicaragua’s iCCM program and demonstrates the value of leveraging program monitoring data for evaluating impact. MINSA and its partners have designed and implemented a strong iCCM program within PROCOSAN, which has increased the use of curative interventions for sick children, reducing infant deaths.

## Acknowledgments

The authors thank government health colleagues at the district (*municipio*) and departmental levels, as well as their colleagues in MINSA who helped design, plan, introduce, scale up, and monitor the program, especially Miguel Velazquez, Emerita Corrales, and Ivania Lainez. The authors also gratefully acknowledge a 2008 evaluation conducted by Johns Hopkins University (Baltimore, Maryland); the U.S. Agency for International Development (USAID) (Washington, DC); and the United Nations Children’s Fund (UNICEF) (New York). Support for data analyses and manuscript writing came from Save the Children USA. The authors thank Yury Higuchi for preparing the map of the study areas. They also express their appreciation to the *brigadistas* who delivered iCCM and to the families who entrusted their sick children to the *brigadistas*’ care.

## Disclaimer.

Authors hold responsibility for the views expressed in the manuscript, which may not necessarily reflect the opinion or policy of the *RPSP/PAJPH* or the Pan American Health Organization (PAHO).

## Funding.

Program support came from Save the Children USA (Fairfield, Connecticut) corporate and private donors and the USAID Basic Support for Institutionalizing Child Survival (BASICS) program. The funders had no role in the study design, data collection and analysis, decision to publish, or preparation of the manuscript.

## Footnotes

^5^ For example, supervisors used well children from growth-monitoring sessions to assess *brigadistas*’ ability to count respiratory rates.

^6^ Summary table.

^7^ https://ideas.repec.org/c/boc/bocode/s426101.html
